# ﻿The correct name for an *Aquilegia* (Ranunculaceae) hybrid of the parentage *Aquilegiaflavescens* × *A.formosa*

**DOI:** 10.3897/phytokeys.220.99170

**Published:** 2023-02-23

**Authors:** Quentin C. B. Cronk

**Affiliations:** 1 Department of Botany, University of British Columbia, 3156-6270, University Blvd., V6T 1Z4, Vancouver, BC, Canada University of British Columbia Vancouver Canada; 2 Beaty Biodiversity Museum, University of British Columbia, Vancouver, V6T 1Z4, BC, Canada University of British Columbia Vancouver Canada

**Keywords:** *Aquilegia* × *miniana*, columbines, hybridization, linear discriminant analysis, Ranunculales, Wells diagram

## Abstract

Aquilegia×miniana (J.F.Macbr. & Payson) Cronk, **hybr. & stat. nov.** is the correct name for the hybrid *Aquilegiaflavescens* S.Watson × A.formosaFisch. & DC.var.formosa. In 1916 Payson and Macbride, while exploring the mountains of Idaho, found populations of *Aquilegia* that were pink in flower colour and appeared intermediate between the yellow-flowered *A.flavescens* and red-flowered *A.formosa*. They named these plants A.flavescensvar.miniana J.F.Macbr. & Payson. There has been uncertainty over whether their type collections (in GH, RM, MO, US, E, CM, CAS, NY) do indeed represent hybrids or pink-flowered morphs of *A.flavescens*. Using a Wells diagram, the holotype (in the Gray Herbarium of Harvard University) is shown to be intermediate, allowing its identification as a clear hybrid. However, some of the isotype material is indistinguishable from *A.flavescens*. The holotype matches material from British Columbia that has been determined to be of hybrid origin using molecular and morphological data. A.flavescensvar.miniana J.F.Macbr. & Payson is, therefore, an available name for the hybrid, which is here raised to the status of hybrid binomial.

## ﻿Introduction

*Aquilegiaflavescens* and *A.formosa* are an ecologically separated species pair. Aquilegiaformosavar.formosa is a widespread taxon of forest margins and light forest shade from sea level to montane forest, common across western North America from Utah to Alaska. By contrast, *A.flavescens* is a meadow plant of subalpine and alpine meadows, restricted to the western cordilleras from Utah to Alberta and British Columbia. The flowers of both species are strikingly different: *A.formosa* has bright red sepals, whereas *A.flavescens* has yellow (although pink-flowered morphs very rarely occur). Other distinguishing characters are more subtle. In *A.formosa*, the sepals are more tapered, the petal spurs straight and long (rather than incurved and slightly shorter), while the petal blades are small and yellow (rather than larger and paler) and the stamens are strongly exserted (less so in *A.flavescens*). In mountain regions where the two species co-occur, hybridization takes place. Hybrid populations between *A.flavescens* and *A.formosa* are very familiar to botanists in British Columbia (Canada), Idaho and Washington State (USA). No hybrid binomial up until now has been given for this despite its familiarity.

## ﻿History

The hybrid was apparently encountered by Macbride and Payson (1917) during their important early botanical exploration of the mountains of Idaho in 1916 (Williams 2017). On *Aquilegia*, they write: “It has been recognized for some time that *A.flavescens* and *A.formosa* merge in the territory where their ranges join. As a result, there exist many intermediate forms, which cannot be definitely referred to by either of these species. One such state has evolved in central Idaho. There, in many localities, it entirely replaces the typical form of the species, so apparently, it has acquired a certain degree of stability. This form is similar to *A.flavescens* except that the sepals are salmon-color or flushed with pink. This color modification is striking and extremely beautiful, well worth it would seem, of varietal recognition.”

Accordingly, they provided a new varietal name: A.flavescensS.Watsonvar.miniana J.F.Macbr. & Payson and provided an extensive series of type material from localities in central Idaho as exemplars. If these are regarded as hybrids, then the treatment as a variety of one of the parents is clearly unsatisfactory in modern usage. However, the varietal treatment clearly indicates Macbride and Payson’s view that these populations, although intermediate, were closer to *A.flavescens* than to *A.formosa* (Macbride and Payson 1917). As hybrids, we would now see this in terms of introgression in the direction of *A.flavescens*.

A complication was outlined by Whittemore in his Flora of North America treatment of *Aquilegia* (Whittemore 1997). He noted that in natural populations, in addition to the pink-flowered hybrids, some pink-flowered plants are indistinguishable from the “pure” *A.flavescens* on morphological grounds (besides colour) and must therefore be considered a colour variant of *A.flavescens*. He considered that the type of A.flavescensvar.miniana was one of these: “*Aquilegiaflavescens* sometimes forms hybrid swarms with A.formosavar.formosa, which grows at lower elevations through much of its range. Intermediate specimens having pinkish-red flowers and petal blades 5–6 mm are occasionally found where these species grow together. The name A.flavescensvar.miniana has sometimes been mistakenly applied to these intermediates, but the type of var. miniana is a typical, pink-sepaled plant of *A.flavescens*” (Whittemore 1997). However, the specimens annotated by Whittemore do not include the holotype (GH) but are instead isotype material (MO). Furthermore, MacBride and Payson are explicit in the protologue that they considered the populations from which they gathered the type material to be intermediate (“intermediate forms which cannot be definitely referred to either of these species”), with the implication of hybrid origin. In a recent study, Groh and Cronk (2020) undertook an extensive morphometric examination of herbarium material (including Macbride and Payson’s type material). They presented evidence that the type population was (at least partly) of hybrid origin (Groh and Cronk 2020) but introgressed in the direction of *A.flavescens*, with (as Whittemore pointed out) some specimens close or indistinguishable from *A.flavescens*. Although not explicitly mentioned in that paper, the holotype (GH) was found to be intermediate and, therefore, an available name for *A.flavescens* × *A.formosa*. This paper aims to illustrate the morphological intermediacy of the holotype and, therefore, the hybridity of A.flavescensvar.miniana and raise the name to hybrid binomial status to provide a convenient name for this widespread and conspicuous plant.

## ﻿Methods

The complete list of type material (13 specimens) that has been examined in this study and a previous study (Groh and Cronk 2020) is as follows (summarised in Table 1):

**Table 1.** Wells Index for type specimens of A.flavescensvar.miniana J.F.Macbr. & Payson, compared with reference populations of pure species (see Fig. 2). Linear discriminant analysis (LDA) scores are given for comparison. Asterisks indicate that the specimen is abutting (*) or within (**) the range of *A.flavescens*.

Locality	Date	Coll. No.	Herbaria [type]	Wells index	LDA
Mt Kobau (BC) [*flavescens*]		MK1-MK20	UBC	0.9460-1.1535	-4.0443 - -7.3611
Roberts Lake (BC) [*formosa*]		RC1-RC20	UBC	0.1739-0.4285	3.8729 - 7.3717
Challis Creek	July 19, 1916	3326	GH [HOLO.]	0.7726	-1.5934
			RM [ISO.]	0.8217	-2.1263
			MO (#1) [ISO.]	0.8127	-2.4344
			MO (#2) [ISO.]	0.9070*	-3.6754*
			US [ISO.]	0.7306	-0.7466
			E [ISO.]	0.7958	-2.1444
			CM [ISO.]	0.7114	-0.7579
			CAS [ISO.]	0.8250	-2.1715
			NY [ISO.]	0.7438	-1.3846
Bonanza	July 28, 1916	3487	RM [PARA.]	0.7989	-1.6663
Sawtooth Peaks	Aug. 9, 1916	3692	RM [PARA.]	0.9287*	-4.2024**
		3692	CM [PARA.]	0.9704**	-4.5946**
Smoky Mts.	Aug. 13, 1916	3751	RM [PARA.]	0.7989	-1.9128

IDAHO. Custer Co.: Stream bank in shade, Challis Creek, July 19, 1916, J.F.Macbride & E.B.Payson 3326 (GH [holo.], RM [iso.], MO 2 ex [iso.], US [iso.], E [iso.], CM [iso.], CAS [iso.], NY [iso.]); rocky, protected rocky hillside, Bonanza, July 28, 1916, J.F.Macbride & E.B.Payson 3487 (RM [para.]). Blaine Co.: along alpine brook, Sawtooth Peaks, Aug. 9, 1916, J.F.Macbride & E.B.Payson 3692 (RM [para.], CM [para.]); crevices of granitic rock, Smoky Mts., Aug. 13, 1916, J.F.Macbride & E.B.Payson 3751 (RM [para.]).

### ﻿Wells ordination

To complement the linear discriminant approach previously established (Groh et al. 2019; Groh and Cronk 2020), a Wells ordination approach has been used here (Wells 1980). The Wells method has been widely used in studies of hybridization, and for examples and discussion, see Christensen and Dar (1997); Diaz Lifante and Andres Camacho (2007); Little (2004). The morphometric data is taken from herbarium specimens and is previously generated and described by Groh et al. (2019) and Groh and Cronk (2020). Briefly, the following seven quantitative characters have been used: (a) anther exsertion; (b) corolla width; (c) petal lamina length; (d) petal spur length; (e) petal lamina width; (f) sepal length; and (g) sepal width. To avoid transport stress on the type specimens, all measurements were made using high-resolution images provided via JSTOR Global Plants (https://plants.jstor.org). The holotype material was later examined physically in GH.

In addition to the type material (13 Idaho specimens), two parental reference populations were chosen. For *A.flavescens*, a sample of 20 plants from the Mt Kobau population in southern British Columbia (BC) was used. It is morphologically typical of the species, and no *A.formosa* is known nearby. For *A.formosa* 20 individuals were sampled from a population at Roberts Lake, Vancouver Island, BC. These are typical of the species and allopatric to *A.flavescens*. Both parental populations have been shown to be pure species by molecular and morphological methods, with no evidence of hybridity (Groh et al. 2019; Groh and Cronk 2020).

The Wells method requires the construction of parental endpoints for analytical purposes by taking extreme values for each character associated with each species. Then the Euclidian distance is calculated between these endpoints and all the specimens in the analysis. Each specimen is then triangulated onto a plot by means of its distance from each of the species’ endpoints and the distance between the endpoints themselves (Wells 1980). The raw measurements were log-transformed and ranged between 0 and 1. Weights were then applied for each character depending on their discriminatory power between the putative parents, calculated as the ratio of the mean of the within parent standard deviations (S_mp_) to the standard deviation of both parents pooled (S_t_), i.e., Wi = 1–S_mp_/S_t_ (Wicksell and Christensen 1999). For comparison, a linear discriminant analysis (LDA) on the untransformed data was performed using PAST 4 (Hammer et al. 2001).

### ﻿Data resources

The data underpinning the analysis reported in this paper are deposited in the Dryad Data Repository at https://doi.org/10.5061/dryad.79cnp5j0f.

## ﻿Results

### ﻿The holotype and other type specimens

Four collection numbers (Challis Creek 3326, Bonanza 3487, Sawtooth Peaks 3692, and Smoky Mts 3751) are cited in the protologue, with the Challis Creek gathering the main one on which the name is founded. Numerous duplicates were taken in the Challis Creek gathering. Still, only the single specimen at the Gray Herbarium (GH) is singled out, and it is the only specimen specifically designated as “Type”. It must therefore be considered the holotype. The others are from the same gathering (3326) and, therefore, isotypes (although they likely represent different genetic individuals).

### ﻿Identification of the holotype

**Figure 1.**
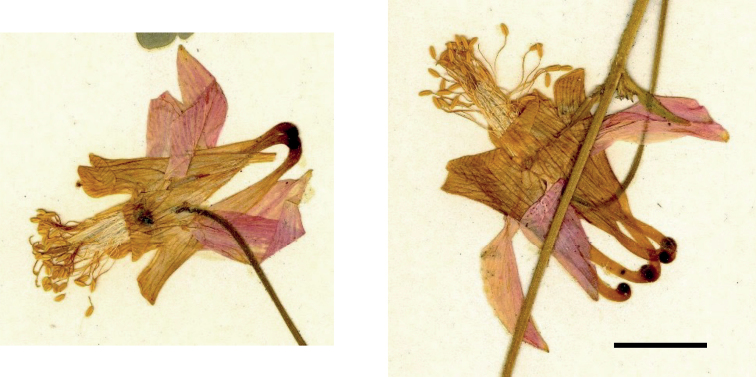
The two flowers preserved as part of the holotype specimen (GH). Scale bar: 1 cm.

The holotype (Fig. 1) cannot satisfactorily be identified as *A.flavescens*. The petal blades are c. 6 × 6 mm, whereas in *A.flavescens*, they are typically larger (8 × 8 mm), and in *A.formosa*, they are smaller (4 × 4 mm). The anthers of the holotype are strongly exserted, c. 10 mm, a feature of *A.formosa* (12–15 mm), rather than *A.flavescens* (typically 5–8mm). Finally, the flowers still show clear traces of pink coloration (not yellow as is general in *A.flavescens* or red as always in *A.formosa*). These features, taken together, are sufficient to allow the identification of the holotype as a hybrid. It strongly resembles material that has been identified as a hybrid by molecular methods in British Columbia in a previous study (Groh et al. 2019).

### ﻿Quantitative morphological intermediacy of the holotype and other type specimens

**Figure 2.**
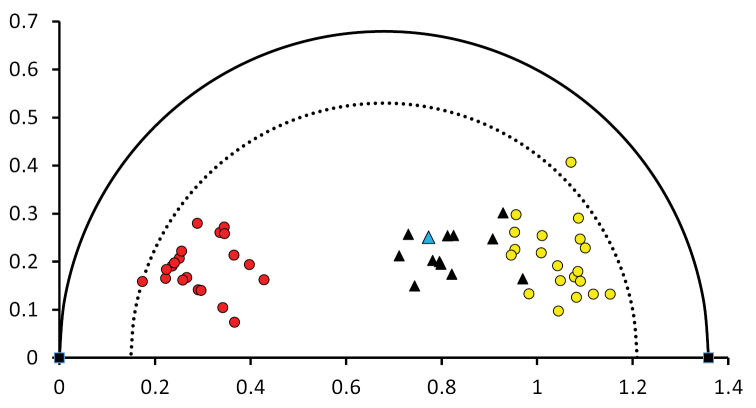
Wells diagram of *Aquilegia* samples. The red circles are the reference population of *A.formosa*, and the yellow circles are the reference population of *A.flavescens*. The black triangles denote type specimens, with the blue triangle indicating the holotype. The outer semicircle connects the two reference extremes on the x-axis, whereas the inner semicircle encloses all the parental specimens (except one outlier). The type specimens occupy an intermediate position, although skewed towards and intergrading with *A.flavescens*. The type specimens are also all within the inner semicircle, satisfying the theoretical expectations of intermediacy. The numerical position on the x-axis is the Wells Index (as given in Table 1).

Quantitative data from the type specimens are summarised graphically in Fig. 2. These results complement the results previously published (Groh and Cronk 2020). The figure distinguishes the holotype from the isotypes and paratypes so that taxonomic conclusions can be drawn. Although the type specimens generally show intermediacy between the two parents, it also appears that certain specimens (from RM and MO) are indistinguishable from *A.flavescens*, as Whittemore correctly pointed out. However, as the other specimens show the intermediacy expected of hybrids, this is indicative of the type of material drawn from hybrid populations, as implied by Macbride and Payson (1917). Moreover, the holotype (GH) is distinctly intermediate between the two putative parents (Fig. 2). Macbride and Payson’s name (A.flavescensvar.miniana) is consequently confirmed as an available name for the hybrid.

### ﻿Nomenclature

Naming a hybrid as a variety of one of its parents is not modern practice, and a hybrid binomial would be more beneficial for this widespread and characteristic hybrid. Accordingly, it is here raised to hybrid status:

#### ﻿ Aquilegia × miniana

Taxon classificationPlantaeRanunculalesRanunculaceae

(J.F.Macbr. & Payson) Cronk, hybr. & stat. nov.

E5F9AE60-7A3E-55CC-B4BA-3F7F7C704D02

A. flavescens var. miniana J.F.Macbr. & Payson, 1917, Contr. Gray Herb., 49: 61. Basionym.

##### Type material.

***Holotype*.** Macbride and Payson 3326 (GH). A hybrid of the parentage: *Aquilegiaflavescens* S.Watson × *A.formosa* Fisch. & DC., with intermediacy between the two parents.

##### Type locality.

In Payson (1918), further details are given of the type locality (Challis Creek) in relation to parental populations: “…Custer County, Idaho. There, near the town of Challis, at an altitude of 1620 meters, was found nearly typical *A.formosa*, while on the slopes of Parker Mountain, about 25 miles away and at an altitude of 2,400 to 2,700 metres, was found nearly typical *A.flavescens*. Intermediate forms were met along Challis Creek between these altitudes.”

It is necessary to discuss an earlier name, *Aquilegiarubicunda* Tidestr. (Tidestrom 1910), collected by Tidestrom in the Wasatch Mountains of Utah. This is also an *Aquilegia* with pink sepals. It has been considered a pink-flowered form of *A.flavescens* (Welsh 1986) and, as such, might represent an earlier name for A.×miniana. However, the type specimen (US) bears no particular similarity to *A.flavescens* or its hybrid with *A.formosa*. Whittemore (1997) considered it to be *A.micrantha*. It might well repay further study, but we can rule out relevance to the hybrid considered here.

## Supplementary Material

XML Treatment for Aquilegia × miniana
